# Effect of Siphon Morphology on the Risk of C7 Segment Aneurysm Formation: A Case-control CFD Study

**DOI:** 10.1007/s00062-024-01394-3

**Published:** 2024-02-28

**Authors:** Ying Wang, Bo Chen, Laixin Song, Yuzhe Li, Ming Xu, Tianxiang Huang, Feiyue Zeng

**Affiliations:** 1 Department of Radiology, Xiangya Hospital, Central South University, No. 87 Xiangya Rd, 410008 Changsha, Kaifu District China; 2 Department of Surgery, School of Clinical Medicine, LKS Faculty of Medicine, The University of Hong Kong, Queen Mary Hospital, Hong Kong SAR, China; 3 Department of Neurosurgery, Xiangya Hospital, Central South University, Changsha, Hunan China; 4 Hunan Provincial Key Laboratory of Clinical Epidemiology, Xiangya School of Public Health, Central South University, Changsha, Hunan China; 5 National Clinical Research Center for Geriatric Disorders, Xiangya Hospital, Central South University, Changsha, Hunan China

**Keywords:** Carotid siphon, Aneurysm formation, Morphological parameters, Computational fluid mechanics, Hemodynamics

## Abstract

**Purpose:**

Tortuosity of the internal carotid artery (ICA) is associated with intracranial aneurysms (IAs). The siphon is the most curved segment of the ICA, but its morphology has controversial effects on IAs. This study aimed to explore the morphometric features of the siphon and the potential hemodynamic mechanisms that may affect C7 aneurysm formation.

**Methods:**

In this study 32 patients with C7 aneurysms diagnosed at Xiangya Hospital between 2019 and 2021 and 32 control subjects were enrolled after propensity score matching. Computed tomography angiography (CTA) images were acquired to measure morphologic features, and then, by combining clinical data, simplified carotid siphon models were constructed, and computational fluid dynamics (CFD) analysis was performed.

**Results:**

The presence of C7 aneurysms was associated with the height of the C4–C6 curved arteries (odds ratio [OR] 0.028, 95% confidence interval [CI] 0.003–0.201; *P* < 0.001). The heights of the C4–C6 curved arteries in the aneurysm group were significantly shorter than those in the control group. The CFD analysis revealed that shorter C4–C6 bends led to greater blood velocity and pressure in the C7 segment arteries.

**Conclusion:**

A shorter C4–C6 bend was associated with distal C7 aneurysm formation, and an elaborate hemodynamic mechanism may underlie this association.

**Supplementary Information:**

The online version of this article (10.1007/s00062-024-01394-3) contains supplementary material, which is available to authorized users.

## Introduction

Intracranial aneurysm (IA) is a common cerebral vascular disease characterized by artery wall outpouching [1]. Almost 85% of IAs develop at the bifurcation of anterior circulating arteries and at the beginning of posterior communicating arteries (PCoAs) [2]. Bouthillier et al. classified the internal carotid artery (ICA) into C1–C7 segments, including C4 (cavernous), C5 (clinoid), C6 (ophthalmic), C7 (communicating) and others [3]. A primary aneurysm in the C7 segment is called the PCoA aneurysm, which ruptures more frequently than other IAs [4, 5].

Morphology and hemodynamics are two important factors that may be linked to aneurysm formation [6, 7]. The carotid siphon is the main segment of intracranial ICA adjacent to C7 and consists of the C4–C6 segments and multiple bends. Blood flow in the intracranial arteries is affected by characteristics of the siphon; however, the relevance of carotid siphon bends in aneurysm formation is still debated. Waihrich et al. discovered that carotid siphons with more acute anterior angles generate more turbulent flow, which contributes to a greater incidence of IA in carotid siphons [8]. Moreover, the high bend curvature of the carotid siphon induces dynamically fluctuating high wall shear stress (WSS) and a wall shear stress gradient (WSSG), which cause damaging vascular wall remodeling and aneurysm initiation [9]. The tortuosity of the ICA is hypothesized to be associated with IA formation. Unexpectedly, another study revealed no link between the morphological characteristics of carotid siphons and the incidence of PCoA aneurysm [10]. Double bend (S-shaped) siphons are more tortuous than V‑shaped siphons. Patients with S‑shaped siphons were found to be less prone to developing IAs [11], which contradicts earlier findings; however, additional studies are needed to determine the effect of siphon bends on aneurysm formation.

The present study was designed to explore bend-associated hemodynamic mechanisms that may trigger distal aneurysm formation by extracting the morphologic features of proximal siphons, building simplified geometry models, and performing computational fluid dynamics (CFD) analysis.

## Methods

### Patient Selection and Matching

This study was approved by the Institutional Review Board of Xiangya Hospital Central South University (202103613). Written informed consent was obtained from each patient.

**Fig. 1:**
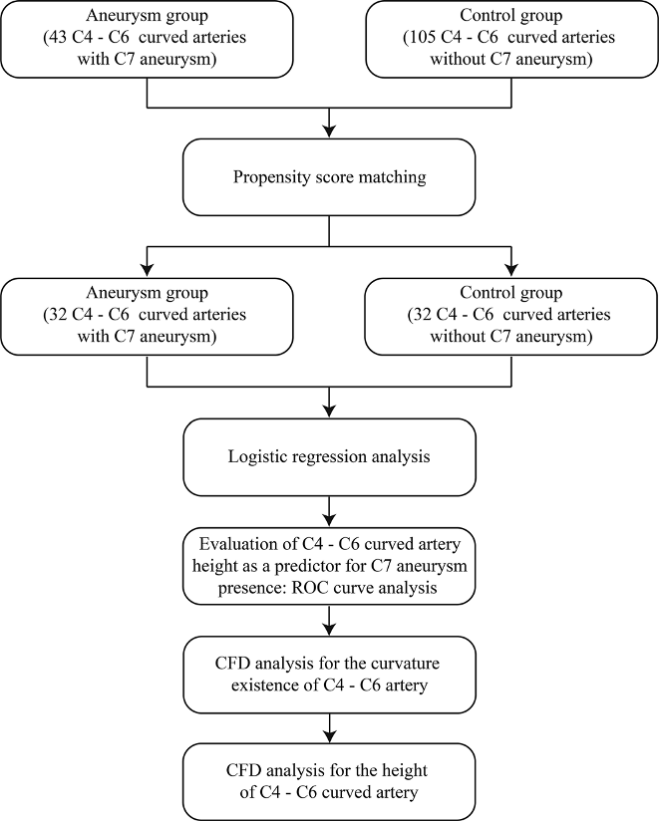
Flow diagram of the study design

In this study, we enrolled patients with unilateral C7 segment (PCoA) aneurysms confirmed by computed tomography angiography (CTA) from December 2019 to July 2021 in the neurosurgery department of Xiangya Hospital Central South University. The exclusion criteria were as follows: 1) aneurysm associated with arteriovenous malformation or moyamoya disease; 2) dissecting aneurysm; 3) mycotic aneurysm; 4) recurrent aneurysm treated with surgical clipping or interventional embolization; 5) aneurysm > 10 mm, which may affect the morphology of the parent vessel; 6) severe carotid artery stenosis (> 70%, according to the North American Symptomatic Carotid Endarterectomy Trial method) and 7) treatment with mechanical thrombectomy in the setting of acute ischemic stroke [12, 13]. The control subjects were people who underwent CTA screening and were confirmed to be without aneurysms during the same period. Of the 148 patients enrolled in our research, 43 patients with unilateral C7 segment aneurysms were assigned to the aneurysm group, and 105 patients without aneurysms were assigned to the control group. Clinical baseline data, including age, sex, hypertension, diabetes, hyperlipidemia, smoking, and alcoholism, were collected. We used 1:1 propensity score matching (PSM) to balance clinical baseline features between the two groups. The matched patients included 32 patients with C7 segment aneurysms and 32 controls. A flow diagram of the analysis is displayed in Fig. 1.

### CTA Performance

The CTA was performed on a 320 multislice computed tomography (CT) scanner (Aquilion ONE, Toshiba Medical Systems, Otawara, Japan). Nonionic iodinated contrast medium (370 mg/dose; Ultravist 370; Bayer Schering Pharma, Berlin, Germany) was injected intravenously through an automatic injector (5 ml/s; Stellant system, MEDRAD, Berlin, Germany). Bolus tracking was used to regulate the time when the contrast agent arrived in the carotid artery. Then, axial data were obtained at 0.5 mm intervals and transferred to diagnostic imaging analysis software (VitreafX, Version 3.1.0; Toshiba, Tokyo, Japan) for postprocessing and further study.

### Morphological Features

The 3D vessels were reconstructed using CTA DICOM axial data in Mimics research software (version 21.0, Materialise, Inc., Leuven, Belgium). Morphological data were independently measured by two observers skilled in neuroimaging using 3‑Matic research software (version 13.0; Materialise), and the values were averaged. The two observers were blinded to patient information except for the ID numbers in the electronic medical record system. If significant disagreements (> 0.1 cm) occurred between the two observers, the morphological data were submitted to and measured by a third observer to exclude errors. In addition, Bland-Altman analysis was used to verify the consistency between the observers.

### Measurements

**Fig. 2:**
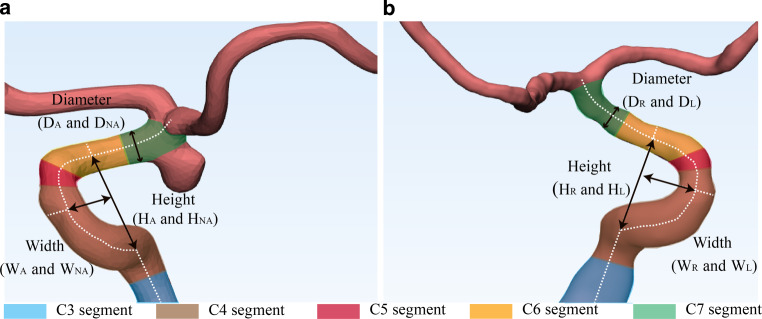
Schematic drawings of the morphologic features measured in our study. **a** and **b** Schematic drawing showing the height and width of the C4–C6 bend and the diameter of the C7 segment in aneurysm patients (**a**) and control patients (**b**). *A* aneurysmal side in aneurysm patients, *NA* non-aneurysmal side in aneurysm patients, *R* right side in control subjects, *L* left side in control subjects, *D* diameter, *W* width, *H* height

We measured three morphologic variables of the bilateral ICA, including the height and width of the C4–C6 curved segments and the diameter of the C7 segment. The measuring methods are shown in Fig. 2. In the aneurysm group, we measured the aneurysmal (_A_) and nonaneurysm (_NA_) sides, and in the control group, we measured the left (_L_) and right (_R_) sides. The center line of the artery was used as the marked line for the measurement of the siphon height and width. The height (H) was defined as the distance between the C6 segment and the horizontal section of the C4 segment. The width (W) was defined as the anterior-posterior distance between the origin and terminal parts of C4. The diameter (D) of arteries was measured at the initial part of the C7 segment. The height-to-width ratio (HWR) of C4–C6 curved arteries was calculated.

### CFD Analysis

We reconstructed 3D ICA models in Mimics research software (version 21.0, Materialise Inc., Leuven, Belgium) for all cases. To investigate the role of the C4–C6 bend in C7 segment hemodynamics, we virtually modified the curved artery into a straight artery by mask editing at the multiple consecutive horizontal planes. The 3D ICA models with and without bends were exported to the ANSYS Workbench Fluent software (version 20.1, ANSYS Inc., Pittsburgh, PA, USA) for CFD analysis.

To further investigate the role of C4–C6 curved artery height in C7 aneurysm formation, we constructed a group of simplified 3D ICA models with variable bend heights based on real clinical data (Fig. 5a). The width of the curve was set to 1.0 cm (based on the range of W_R_, W_L_, W_A_, and W_NA_, 0.66–1.66 cm). The height of the bends was varied from 0.6 cm to 1.4 cm (based on the range of H_R_, H_L_, H_A_, and H_NA_, 0.32–1.36 cm). The diameters of the ICA, ACA, and middle cerebral artery (MCA) were set to 3.5 mm, 2.0 mm, and 2.8 mm, respectively [14]. The parent-daughter angles of ICA-ACA and ICA-MCA were set to 117° and 135°, respectively [14]. The model was divided into 0.15 mm tetrahedral meshes with a 5-layer boundary layer mesh near the fluid boundaries. The blood flow was defined as a laminar and incompressible Newtonian fluid, with a density of 1050 kg/m^3^ and viscosity of 0.0032 kg/(m*s) [12]. The inlet boundary of the model was defined as a pulsatile velocity inlet (Supplemental Method 1) [15]. The outlet boundaries of the model were defined as 0 Pa pressure [16]. The time step was set to 0.003 s.

**Fig. 3:**
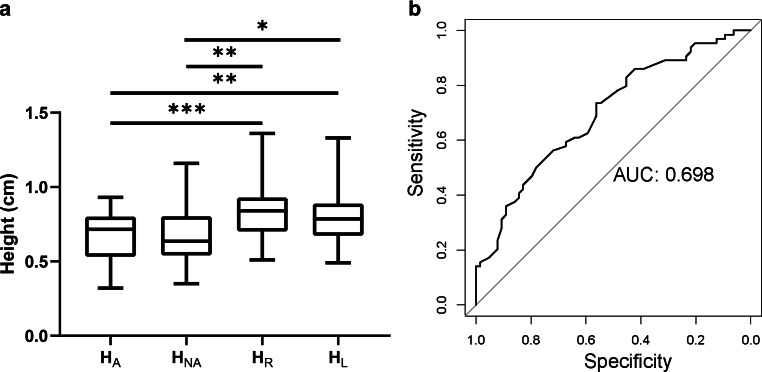
Comparison of C4–C6 bend heights between the aneurysm and control groups. **a** Patients with C7 aneurysms had shorter C4–C6 bend heights than control subjects, **b** ROC analysis showed that C4–C6 bend heights could predict patients with C7 aneurysms; AUC = 0.698. *AUC* area under the curve

### Statistical Analysis

Data were analyzed using SPSS software (version 26.0, IBM Inc., Armonk, NY, USA). Normality of the data distribution was examined by the Shapiro-Wilk normality test. To compare continuous variables between the two groups, the two-tailed t test (for normal distribution) and Mann-Whitney test (nonnormal distribution) were used. Analysis of variance (ANOVA) (normal distribution) and the Kruskal-Wallis test (nonnormal distribution) were used for comparisons among multiple groups. In addition, we performed the χ^2^-test and Fisher’s exact test to compare categorical variables. Multivariate logistic regression was used to identify independent risk factors. To predict which bend was prone to developing a C7 aneurysm, receiver operating characteristic (ROC) analysis was conducted. A *P* value < 0.05 was considered statistically significant.

## Results

### Demographics

The clinical data of the two groups before and after PSM are summarized in Table 1. Of the 32 subjects in the control group, 10 were males and 22 were females, with a mean age of 54.78 ± 6.55 years. Among the 32 patients in the aneurysm group, 10 were males and 22 were females, with a mean age of 53.88 ± 6.06 years, 9 patients had ruptured aneurysms, and 23 patients had unruptured aneurysms.

**Table 1:** Summary of clinical data before and after PSM

	Before PSM			After PSM		
	Control group (*n* = 105)	Aneurysm group (*n* = 43)	*P* value	Control group (*n* = 32)	Aneurysm group (*n* = 32)	*P* value
Age (years)	54.17 ± 8.99	54.79 ± 5.59	0.614	54.78 ± 6.55	53.88 ± 6.06	0.568
Male (%)	77 (73.3)	10 (23.3)	< 0.001	10 (31.3)	10 (31.3)	1000
Hypertension (%)	86 (81.9)	20 (46.5)	< 0.001	21 (65.6)	20 (62.5)	0.794
Diabetes (%)	10 (9.5)	3 (7.0)	0.859	3 (9.4)	3 (9.4)	1000
Hyperlipidemia (%)	18 (17.1)	1 (2.3)	0.014	1 (3.1)	1 (3.1)	1000
Smoking (%)	45 (42.9)	5 (11.6)	< 0.001	5 (15.6)	5 (15.6)	1000
Alcoholism (%)	40 (38.1)	2 (4.7)	< 0.001	2 (6.3)	2 (6.3)	1000
Height of siphon (cm)	0.80 ± 0.20	0.70 ± 0.19	< 0.001	0.80 ± 0.19	0.70 ± 0.18	< 0.001
Width of siphon (cm)	1.16 ± 0.22	1.15 ± 0.25	0.646	1.17 ± 0.20	1.19 ± 0.23	0.646
Diameter of siphon (cm)	0.32 ± 0.05	0.29 ± 0.05	< 0.001	0.31 ± 0.05	0.29 ± 0.06	0.057

### Morphological Features of Siphons with and without C7 Aneurysms

The two observers showed high agreement in the measurements of carotid siphons (Supplemental Fig. 1). Multivariate logistic regression of morphological factors revealed that the presence of C7 aneurysms was associated with the height of the C4–C6 curved arteries (Table 2, odds ratio [OR] = 0.028, 95% confidence interval [CI] = 0.003–0.201, *P* < 0.001). There were no significant differences between the aneurysm and control groups in terms of width (W_R_, W_L_, W_A,_ and W_NA_), diameter (D_R_, D_L_, D_A,_ and D_NA_), or height/width ratio (HW_R_, HW_L_, HW_A,_ and HW_NA_) (Table 3). The heights of the C4–C6 curved arteries (H_A_ and H_NA_) in the aneurysm group were significantly smaller than those (H_R_ and H_L_) in the control group; however, we did not find any difference in height between the aneurysm side and the non-aneurysm side (Table 3 and Fig. 3a). The ROC curve showed that the height of the C4–C6 curved arteries could accurately predict the presence of C7 aneurysms (area under the curve [AUC] = 0.698) (Fig. 3b). The optimal threshold was 0.785 cm, with 73.4% sensitivity and 56.2% specificity.

**Table 2:** Multivariate logistic regression analysis of morphologic features of carotid siphons associated with C7 segment aneurysm

	OR	95% Cl	*P* value
Height	0.028	0.003–0.201	< 0.001
Width	2.44	0.347–17.799	0.371
Diameter	0.004	0.000–17.916	0.202

**Table 3:** Morphologic factors of carotid siphons in the control and aneurysmal groups

		Value		
Variable/group		Mean	Range	*P* value
*Height*				
Control group (*n* = 32)	H_R_ (cm)	0.83	0.51–1.36	0.001
	H_L_ (cm)	0.81	0.49–1.33	
Aneurysm group (*n* = 32)	H_A_ (cm)	0.67	0.32–0.93	
	H_NA_ (cm)	0.68	0.35–1.16	
*Width*				
Control group (*n* = 32)	W_R_ (cm)	1.17	0.75–1.60	0.936
	W_L_ (cm)	1.18	0.80–1.54	
Aneurysm group (*n* = 32)	W_A_ (cm)	1.18	0.66–1.66	
	W_NA_ (cm)	1.20	0.72–1.64	
*Diameter*				
Control group (*n* = 32)	D_R_ (cm)	0.30	0.22–0.39	0.075
	D_L_ (cm)	0.31	0.22–0.40	
Aneurysm group (*n* = 32)	D_A_ (cm)	0.28	0.19–0.45	
	D_NA_ (cm)	0.29	0.19–0.40	
*Height/width ratio*				
Control group (*n* = 32)	HWR_R_	0.73	0.44–1.14	0.208
	HWR_L_	0.68	0.38–1.23	
Aneurysm group (*n* = 32)	HWR_A_	0.59	0.27–1.15	
	HWR_NA_	0.59	0.33–1.17

**Fig. 4:**
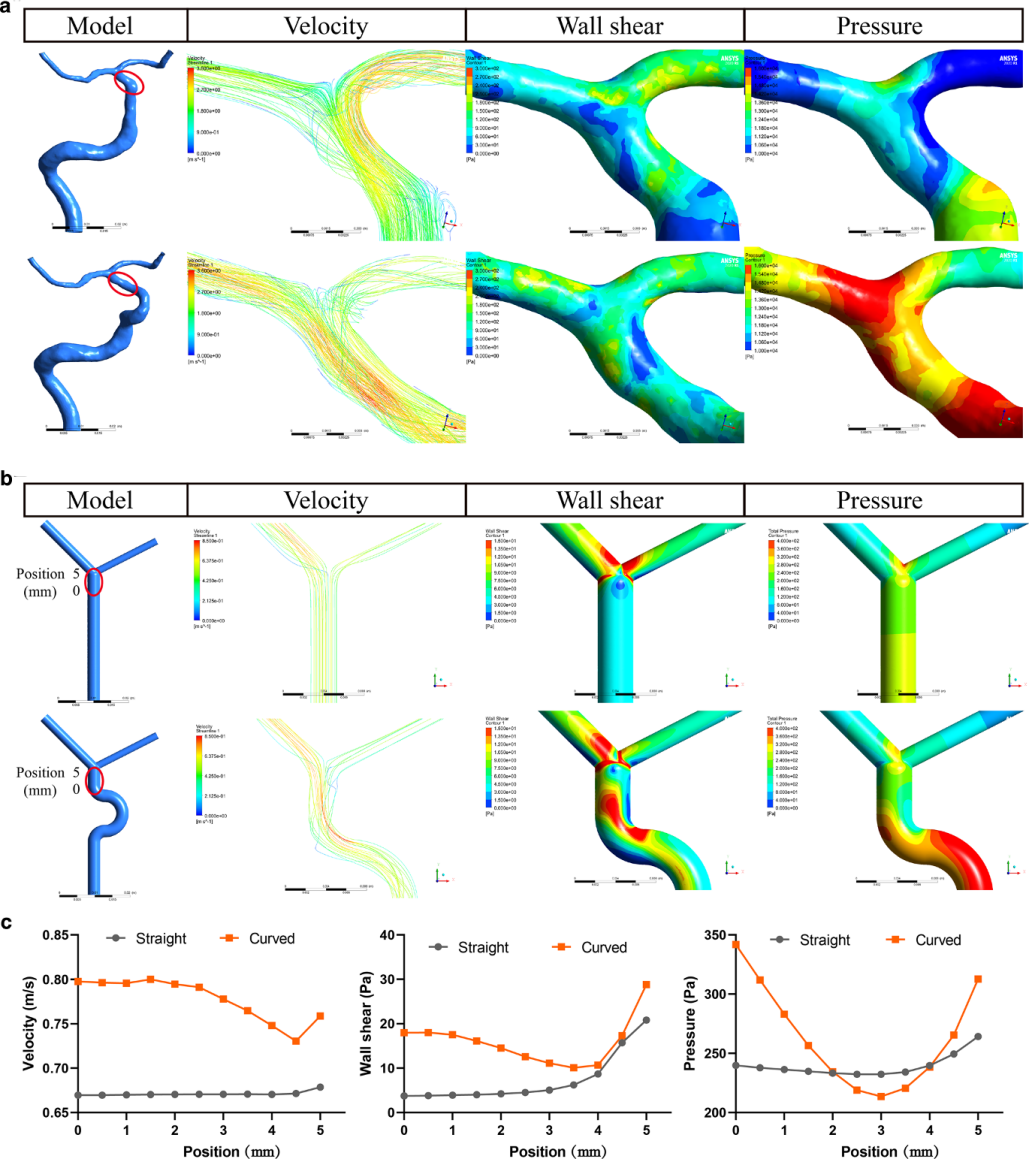
CFD analysis of carotid siphons with and without C4–C6 bends. **a** and **b** Contour graphs of 3D CTA (**a**) and simplified models (**b**) showing the distributions of blood velocity, wall shear and pressure in carotid siphons with (lower row) and without (upper row) the C4–C6 bend. **c** Line graphs of simplified models showing that carotid siphons with C4–C6 bends had greater blood velocity, wall shear and pressure in the C7 segment than siphons without bends. *CFD* computational fluid dynamics, *CTA* computed tomography angiography

### Influence of C7 Aneurysm Rupture on Siphon Morphology

The clinical rupture status data before and after PSM are summarized in Supplemental Table 1. Of the 12 subjects in the unruptured group, 1 was male and 11 were female, with a mean age of 54.08 ± 5.76 years. Among the 12 patients in the ruptured group, 1 was male and 11 were female, with a mean age of 55.58 ± 5.04 years. There were no significant differences in the height (H_A_ and H_NA_), width (W_A_ and W_NA_), diameter (D_A_ and D_NA_), or height/width ratio (HWR_A_ and HWR_NA_) of the C4–C6 curved arteries between the ruptured and unruptured C7 aneurysms (Table 4).

**Table 4:** Comparison of morphologic factors of carotid siphons between ruptured and unruptured C7 segment aneurysms

Variables		Unruptured aneurysm (*n* = 12)	Ruptured aneurysm (*n* = 12)	*P* value
Height	H_A_ (cm)	0.66 ± 0.16	0.73 ± 0.16	0.339
	H_NA_ (cm)	0.71 ± 0.21	0.77 ± 0.17	0.487
Width	W_A_ (cm)	1.08 ± 0.23	1.08 ± 0.26	0.961
	W_NA_ (cm)	1.04 ± 0.29	1.17 ± 0.19	0.186
Diameter	D_A_ (cm)	0.29 ± 0.03	0.27 ± 0.05	0.376
	D_NA_ (cm)	0.28 ± 0.06	0.29 ± 0.05	0.665
Height/width ratio	HWR_A_	0.64 ± 0.23	0.74 ± 0.33	0.590
	HWR_NA_	0.75 ± 0.32	0.68 ± 0.22	0.529

### Influence of Siphon Morphology on the Hemodynamics of C7 Segments

**Fig. 5:**
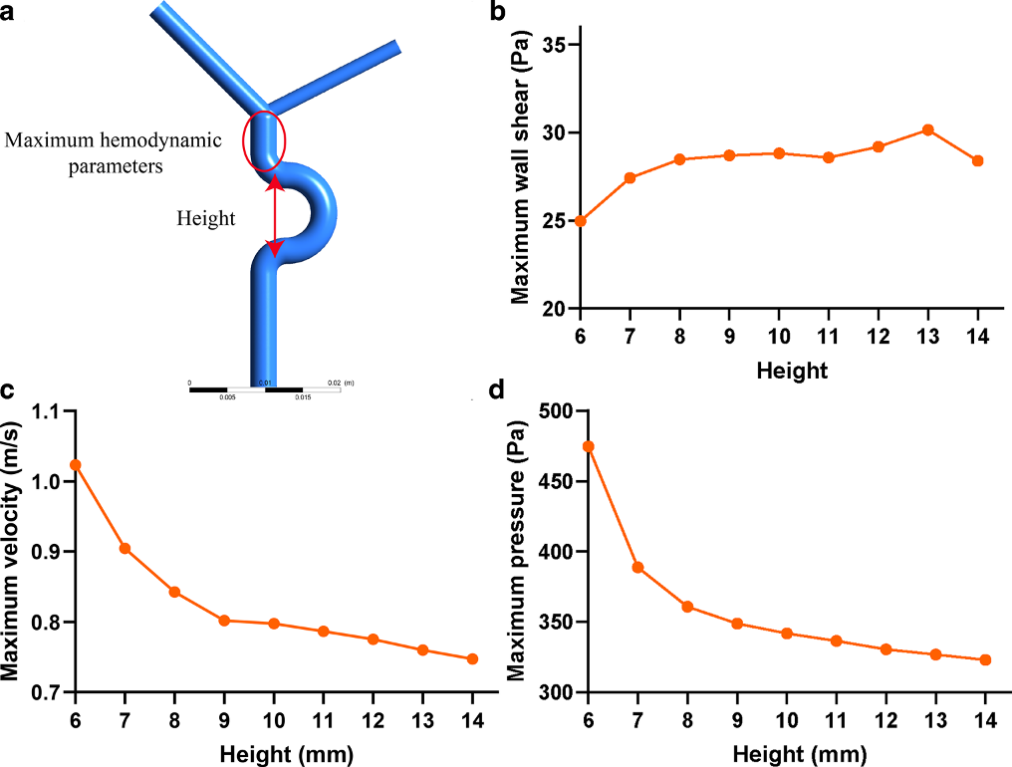
CFD analysis of carotid siphons with different C4–C6 heights. **a** Construction of a simplified model. **b** The maximum WSS of the C7 segments changed little when the C4–C6 bend height changed. **c** and **d** The maximum blood velocity (**c**) and pressure (**d**) in the C7 segments increased when the C4–C6 bend height decreased

The CFD analysis of ICAs with and without C4–C6 bends revealed that the bends increased the blood velocity, wall shear stress, and pressure in C7 segment arteries (Fig. 4a–c). The CFD analysis of simplified 3D ICA models with variable bend heights showed that shorter C4–C6 bend heights led to greater velocity and pressure in C7 segments (Fig. 5c, d). The wall shear stress in the C7 segment exhibited a less pronounced change when the bend height was changed (Fig. 5b).

## Discussion

Vascular tortuosity has been found to affect the formation, development, and rupture of aneurysms [17, 18]. Previous studies have focused primarily on the tortuosity of the whole ICA in patients with IAs. The hemodynamics are primarily influenced by the morphology of the proximal vessels. Little is known about the specific morphology of the siphon. The effect of arterial tortuosity on adjacent distal aneurysm formation also remains unclear. This study aimed to investigate the effect of C4–C6 morphology on C7 aneurysm formation. We observed a high prevalence of C7 aneurysms in siphons, especially when the height of the C4–C6 curved arteries was shorter. The CFD analysis revealed that shorter C4–C6 bends led to greater blood velocity and pressure in the C7 segments.

Early studies have identified relationships between vascular tortuosity and aneurysms [19], including thoracic aneurysms [20], coronary aneurysms [21], and splenic artery aneurysms [22]. Intracranial aneurysms can be affected by the tortuosity of extracranial or intracranial vessels [23, 24]. Vascular tortuosity is usually evaluated by the tortuosity-related length, triangular index, angle metric sum, angle distance product, and other parameters [18]. Anterior communicating artery (ACoA) aneurysms are among the most common aneurysms. Tortuosity of the anterior cerebral arteries increases the risk of ACoA aneurysm development [18]. In patients with basilar artery (BA) aneurysms, the tortuosity index of the BA is significantly elevated [25]. Middle cerebral artery (MCA) aneurysms were also found to be associated with MCA tortuosity [26].

As the last segment of the extradural ICA, the siphon directly affects intracranial blood flow. We believe that C7 aneurysms may be associated with the morphology of the siphon. In our study, we found that C7 aneurysms were commonly located in siphons with a shorter height of the C4–C6 curved arteries. Similarly, Waihrich et al. reported that the anterior knee angle of siphons is related to the formation of intracranial aneurysms located post-siphon through the ICA [8]. Lauric et al. showed that the high bend curvature of the carotid siphon could induce destructive vessel wall remodeling and siphon aneurysm initiation [9]; however, one study found no association between the morphological characteristics of the carotid siphon and the occurrence of PCoA aneurysm [10]. Patients with S‑shaped siphons, which are more torturous than V‑shaped siphons, were found to be less prone to developing IAs [11]. This contradiction may be attributed in part to differences between the studies in the aneurysm position (C4, C5, C6, or C7), morphological variables, and other factors.

In our study, there was no significant difference in the height of the aneurysm side of the ICA compared to the contralateral side. A similar trend was also observed in the control group. Previous studies have reported that approximately 20–30% of patients with IA have multiple aneurysms [2, 27]. The PCoA was frequently associated with multiple IAs and was found to be an independent predictor of rupture in multiple IAs [28]. Mirror IAs, a subtype of multiple IAs, were primarily found in the PCoA, followed by the MCA [29, 30]. The morphology and size of mirror IAs vary between the bilateral corresponding arteries, suggesting that the formation of mirror IAs may occur at different stages. Patients with ruptured aneurysms are at risk of developing a new IA, the frequency of which ranges from 0.37% to 4.13% per patient-year [2]. Roethlisberger et al. reported that patients with a ruptured PCoA aneurysm were more likely to harbor a mirror PCoA aneurysm [31]. We hypothesized that patients with PCoA aneurysms and non-aneurysmal ICAs may be at risk of developing mirror aneurysms; however, further prospective studies are needed to confirm this hypothesis.

Tortuosity reportedly increases the risk of aneurysm rupture [17, 23]. The relationship between bending and rupture may be influenced by various factors, such as the inflow angle. Higher inflow angles of sidewall type aneurysms can increase the risk of rupture, as they allow more blood to flow into the aneurysm and flood the dome. We did not find a significant difference in tortuosity between unruptured and ruptured aneurysms. Several authors have reported that increased tortuosity may reduce the risk of aneurysm growth [32]. An increase in the tortuosity of the ICA can decrease the inflow angle [24, 33], which might explain why high ACA tortuosity could protect aneurysms from rupture [34]. Furthermore, the shape of an aneurysm can also impact the inflow angle and the risk of rupture; however, further research is needed to fully understand the correlation between arterial tortuosity and aneurysm rupture.

The hemodynamic mechanism underlying the development of vascular tortuosity in aneurysms remains unclear. A previous study revealed that the high bend curvature of siphons causes dynamic fluctuations in the WSS and WSSG, which can result in damage to the artery wall and the formation of aneurysms in curved sections [9, 19]. We used CFD analysis to identify aneurysm-related hemodynamics in the distal section of the siphon. After comparing the differences before and after virtual C4–C6 bend removal, we observed that the bend caused turbulence and increased the blood velocity, WSS, and pressure in the C7 segment. We also found that a decrease in the height of the C4–C6 bend resulted in greater velocity and pressure in the C7 segment. Previous studies have confirmed that changes in hemodynamic parameters can contribute to the formation of aneurysms. Meng et al. reported that a combination of high WSS and WSSG induces aneurysm initiation in the area of accelerating flow [35]. High WSS could trigger a mural cell-mediated pathway that predisposes arterial walls to small aneurysm formation and growth [36]. In addition, our previous study revealed that initial intracranial aneurysm sites are characterized by high-pressure regions [12]. Based on these findings, we hypothesized that a shorter C4–C6 bend height may lead to increased turbulence in the distal arteries, resulting in increased maximum blood velocity, pressure, and WSS, ultimately leading to the formation of a distal C7 aneurysm.

This study has several limitations. First, the study was a single-center retrospective case-control study. Additional patients are needed to determine the causal relationship between siphon morphology and IA occurrence. Second, the bias of measurement errors is inevitable due to the complex vascular morphology of some patients, although we took measures to reduce this error. Third, only patients with a C7 segment aneurysm ≤ 10 mm in length were included in the study; therefore, our results may not represent all C7 aneurysms. Fourth, the cervical ICA was not included in our study. The morphological characterization of the ICA is very complicated, and the cervical ICA may influence the results [37].

## Conclusion

Carotid siphons with shorter C4–C6 bends are prone to develop C7 aneurysms. When the height of the C4–C6 bend decreases, the blood velocity and pressure in the C7 segment increase. This hemodynamic change may be related to C7 aneurysm formation.

### Supplementary Information

Supplemental Fig. 1. Agreement between the observers’ measurements assessed using Bland-Altman analysis. The dashed lines represent the 95% limits of agreement.

Supplemental Table 1. Summary of clinical data for aneurysm rupture status.

Supplemental Method 1. Inlet velocity function in computational fluid dynamics (CFD) analysis.

## Data Availability

The data included in the article or supplementary materials or referenced in the article are all available after publication.

## Abbreviations

ACA: Anterior cerebral artery
ACoA: Anterior communicating artery
BA: Basilar artery
CFD: Computational fluid dynamics
CT: Computed tomography
CTA: Computed tomography angiography
D: Diameter
H: Height
HW: Height/width (height to width)
HWR: Height to width ratio
IA: Intracranial aneurysm
ICA: Internal carotid artery
MCA: Middle cerebral artery
PCoA: Posterior communicating artery
PSM: Propensity score matching
ROC: Receiver operating characteristic
W: Width
WSS: Wall shear stress
WSSG: Wall shear stress gradient
