# Supplementary figures and images for: Effect of Siphon Morphology on the Risk of C7 Segment Aneurysm Formation: A Case-control CFD Study
Source: Clin Neuroradiol. 2024 Feb 28;34(2):485–94. doi: 10.1007/s00062-024-01394-3 (PMC11130050; doi:10.1007/s00062-024-01394-3)

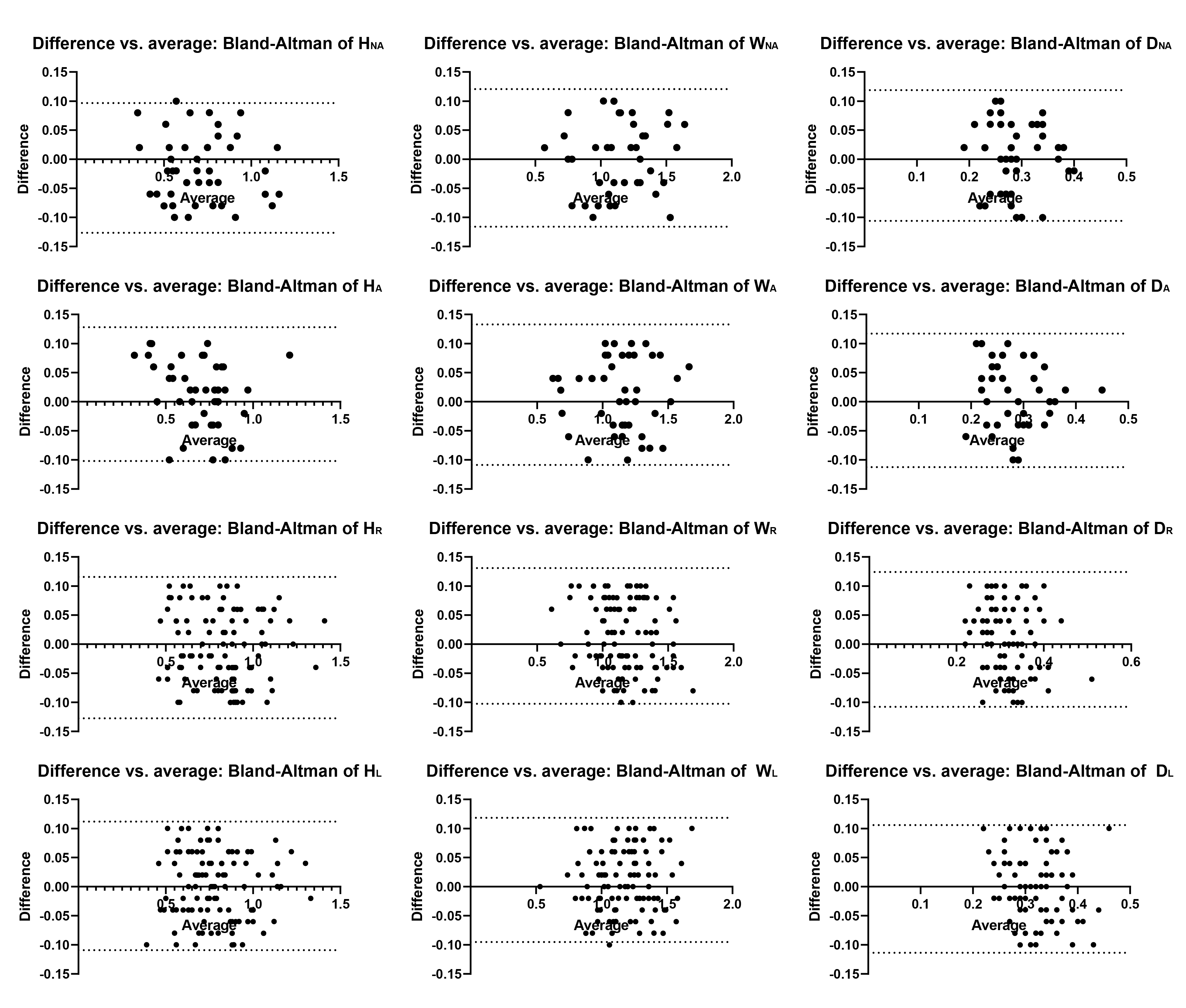

Supplement: Supplementary file 1 — Supplemental Fig. 1. Agreement between the observers’ measurements assessed using Bland-Altman analysis. The dashed lines represent the 95% limits of agreement. [file 62_2024_1394_MOESM1_ESM.tif]
