# Supplementary material for: Effect of Siphon Morphology on the Risk of C7 Segment Aneurysm Formation: A Case-control CFD Study
Source: Clin Neuroradiol. 2024 Feb 28;34(2):485–94. doi: 10.1007/s00062-024-01394-3 (PMC11130050; doi:10.1007/s00062-024-01394-3)
Supplement: Supplementary file 2 — Supplemental Table 1. Summary of clinical data for aneurysm rupture status. [file 62_2024_1394_MOESM2_ESM.docx]

Supplemental Table 1. Summary of clinical data for aneurysm rupture status

|  | Before PSM | | |  | After PSM | | |
| --- | --- | --- | --- | --- | --- | --- | --- |
|  | Unruptured Aneurysm  (*n* = 30) | Ruptured Aneurysm  (*n* = 13) | *P* value |  | Unruptured Aneurysm  (*n* = 12) | Ruptured Aneurysm  (*n* = 12) | *P* value |
| Age (year) | 54.53 ± 5.93 | 55.38 ± 4.87 | 0.652 |  | 54.08 ± 5.76 | 55.58 ± 5.04 | 0.504 |
| Male (%) | 9 (30.0) | 1 (3.3) | 0.112 |  | 1 (8.3) | 1 (8.3) | 1.000 |
| Hypertension (%) | 13 (43.3) | 7 (53.8) | 0.526 |  | 6 (50.0) | 6 (50.0) | 1.000 |
| Diabetes (%) | 2 (6.7) | 1 (7.7) | 1.000 |  | 0 (0.0) | 0 (0.0) | - |
| Hyperlipidemia (%) | 1 (3.3) | 0 (0.0) | 0.505 |  | 0 (0.0) | 0 (0.0) | - |
| Smoking (%) | 5 (16.7) | 0 (0.0) | 0.117 |  | 0 (0.0) | 0 (0.0) | - |
| Alcoholism (%) | 2 (6.7) | 0 (0.0) | 0.340 |  | 0 (0.0) | 0 (0.0) | - |
| Height (cm) | 0.69 ± 0.20 | 0.74 ± 0.16 | 0.324 |  | 0.69 ± 0.18 | 0.75 ± 0.16 | 0.242 |
| Width (cm) | 1.16 ± 0.26 | 1.10 ± 0.23 | 0.737 |  | 1.16 ± 0.26 | 1.12 ± 0.23 | 0.355 |
| Diameter (cm) | 0.29 ± 0.05 | 0.28 ± 0.05 | 0.538 |  | 0.28 ± 0.04 | 0.28 ± 0.05 | 0.859 |

Notes: - indicates the *P* value could not be calculated owing to the existence of 0. However, variables exhibit the same population distribution between the two groups.
