# Supplementary material for: Effect of Siphon Morphology on the Risk of C7 Segment Aneurysm Formation: A Case-control CFD Study
Source: Clin Neuroradiol. 2024 Feb 28;34(2):485–94. doi: 10.1007/s00062-024-01394-3 (PMC11130050; doi:10.1007/s00062-024-01394-3)
Supplement: Supplementary file 3 — Supplemental Method 1. Inlet velocity function in computational fluid dynamics (CFD) analysis. [file 62_2024_1394_MOESM3_ESM.docx]

Supplementary method 1:

Inlet velocity function in computational fluid dynamics (CFD) analysis:

$v_{inlet}\left( t \right)=2761356.325\times t^{12}-13387168.325\times t^{11}+28401303.25\times t^{10}-34610600.75\times t^{9}+26727995\times t^{8}-13606411.075\times t^{7}+4599703.15\times t^{6}-1012965.7225\times t^{5}+137800.46725\times t^{4}-10242.239375\times t^{3}+277.9283166825\times t^{2}+5.4866904225\times t+0.1185213375 \left( 0\leq t\leq0.8s \right).$
